# Case numbers of acute hepatitis of unknown aetiology among children in 24 countries up to 18 April 2022 compared to the previous 5 years

**DOI:** 10.2807/1560-7917.ES.2022.27.19.2200370

**Published:** 2022-05-12

**Authors:** Janko van Beek, Pieter LA Fraaij, Carlo Giaquinto, Delane Shingadia, Peter Horby, Giuseppe Indolfi, Marion Koopmans, Acute hepatitis study group, Acute hepatitis study group, Hatice Aktas, Stefania Bernardi, Oksana Boyarchuk, Daan Van Brusselen, Isabel Casas, Elio Castagnola, Inês Silva Costa, Vibeke Brix Christensen, Joanne Darke, Koen Vanden Driessche, Björn Fischler, Angelo Di Giorgio, Hans Peter Gröchenig, Szofia Hajósi-Kalcakosz, Paul Henderson, Loreto Hierro, Wolf-Dietrich Huber, Piia Jõgi, Julio Werner Juárez, Tatsuo Koide, Valbona Selimaj Kontoni, Inés Loverdos, Cananzi Mara, Inge Matthijs, Paddy Mcmaster, Fatima Mir, Nicolette Moes, Ana Moreno-Álvarez, Thomas Müller, Felipe Ordonez, Anna Ostrzewska, Paolo Paioni, Ana Mocic Pavic, Joanna Pawłowska, David Pérez Solís, Giada di Pietro, Berta Pujol-Soler, Giusy Ranucci, Friedrich Reichert, Nathalie Rock, Burkhard Rodeck, Noris Pavía Ruz, Hans Salzer, Moinak Sen Sarma, Philip Chak on Sham, Eyal Shteyer, Ermelinda Santos Silva, Kärt Simre, Angeliki Syngelou, Rachel Tayler, Goran Tešović, Eunice Trindade, Christos Tzivinikos, Luís Varandas, Herbert van Wering

**Affiliations:** 1 Department of Viroscience, Erasmus MC, University Medical Center Rotterdam, Rotterdam, the Netherlands; 2 Department of Pediatrics, Erasmus MC-Sophia, University Medical Center Rotterdam, Rotterdam, the Netherlands; 3 Department of Women and Child Health, University of Padova, Padova, Italy; 4 Department of Infectious Diseases, Great Ormond Street Hospital, London, United Kingdom; 5 Pandemic Sciences Institute, ISARIC, University of Oxford, Oxford, United Kingdom; 6 Department Neurofarba, University of Florence, Paediatric and Liver Unit, Meyer Children's University Hospital of Florence, Florence, Italy; 7 The members of the group are listed under Collaborators.

**Keywords:** acute hepatitis, unknown origin, outbreak

## Abstract

An increase of acute hepatitis of unknown aetiology has been reported among children in multiple countries worldwide. With a rapid online survey among hospitals in and outside of Europe, we describe case numbers recorded from 1 January to 18 April 2022 vs the previous 5 years. Of 24 countries that responded, we identified 5/17 European and 1/7 non-European countries with an elevation in probable cases of unexplained acute hepatitis, and severe cases were elevated in five European countries.

An increase of acute hepatitis of unknown origin has been reported among children in multiple countries [1,2]. Up to 3 May 2022, 163 cases have been reported from the United Kingdom (UK), of which 11 received a liver transplantation [3]. By 27 April, according to the European Centre for Disease Prevention and Control (ECDC), approximately 55 cases have been reported from 12 other European countries, 12 cases from the United States, 12 from Israel, and 1 from Japan [4]. The cause of this possible outbreak is unclear, but based on initial assessment, an infectious aetiology is considered. Together, a group of European clinical trial networks and the paediatric gastroenterology–hepatology and infectious diseases societies set up a rapid online survey among members to assess the extent and geographical distribution of the suspected outbreak from 1 January until 18 April 2022 in comparison to the incidence in the previous 5 years.

## Online survey and case definitions

The European clinical research network of infectious diseases (ECRAID), Penta–Child Health Research, International Severe Acute Respiratory and Emerging Infection Consortium (ISARIC), European Society for Paediatric Infectious Diseases (ESPID) and European Society for Paediatric Gastroenterology Hepatology and Nutrition (ESPGHAN) developed a survey to collect aggregated data on the number of paediatric cases of acute hepatitis of unknown aetiology during the period from 2017 until 18 April 2022, stratified by year. Participating hospitals were asked to report the number of possible, probable, and severe possible and probable cases according to the case definitions (Box). The online survey was sent out by email to over 3,000 network members working for an unknown number of hospitals on 19 April 2022. All questionnaire responses submitted until 25 April 2022 were included in the analysis.

Box **Case definitions^a^ for acute hepatitis of unknown aetiology, on 19 April 2022**

• Possible case: an individual with acute hepatitis up to 16 years of age AND with a serum transaminase > 500 IU/L, in which diagnostic tests for hepatitis A through E viruses are negative or undetectable, or have not yet been completed.

• Probable case: an individual with acute hepatitis up to the age of 16 years AND with a serum transaminase > 500 IU/L, in which hepatitis A through E virus infections have been excluded.

• Severe possible or probable cases: a possible or probable case with acute liver failure (INR > 2.0).

^a^ Case definitions were based on those by the World Health Organization [1] and United Kingdom Health Security Agency [6].

The case number for 2022 was assigned ‘elevated’ if the number of cases in 2022 (minimum of three absolute cases, corrected for the incomplete year) per hospital was at least three times higher than the mean number of cases in the previous 5 years (arbitrary threshold). 

## Reporting of cases

We received responses from 52 hospitals (of which 13 were liver transplantation centres) in 17 European and 7 non-European countries; four hospitals reporting an unknown number of possible, probable, and severe cases in 2022. Of these 52 hospitals, 20 European and four non-European hospitals reported baseline data for the complete previous 5 years, 17 European and two non-European hospitals reported partial background data (data for at least 1 previous year) and no baseline data was reported by eight European and 1 non-European hospital. Possible cases (n = 49) were reported in Belgium (n = 3), Denmark (n = 3), Greece (n = 1), Hungary (n = 2), Poland (n = 7), Spain (n = 1), the UK (n = 26), Guatemala (n = 1), India (n = 3), and Israel (n = 2) by 13 hospitals in 2022 (Table). None of these 13 hospitals reported an elevated number of possible cases in 2022 compared with the previous 5 years (n = 6) or did not report background data (n = 7). Hospitals in the UK reported a higher number of possible cases in 2022 compared with hospitals in other countries.

**Table:** Numbers of reported possible, probable, and severe cases of acute hepatitis of unknown aetiology among children aged 16 years and under, European (n = 17) and non-European (n = 7) countries, 1 January−18 April 2022

Country	Reporting hospitals (n = 52)	Possible cases (n = 49)	Probable cases (n = 111)	Possible or probable severe cases (n = 36)
European				
Austria	4	0	0	0
Belgium	4	3	0	0
Croatia	2	0	1	0
Denmark	1	3	4	2
Estonia	1	0	0	0
Germany	3	0	1	1
Greece	1	1	0	0
Hungary	1	2	0	0
Italy	7	0	12^a^	5^a^
Poland	1	7	0	5^a^
Portugal	4	0	3	0
Spain	6	1	9^a^	5^a^
Sweden	1	0	4^a^	4^a^
Switzerland	2	0	1	0
Netherlands	1	0	2	1
United Kingdom	5	26	37^a^	5^a^
Ukraine	1	0	6^a^	0
Non-European				
Colombia	1	0	18	4
Guatemala	1	1	0	0
Hong Kong	1	0	0	0
India	1	3	3	3
Israel	1	2	5^a^	0
Mexico	1	0	0	0
United Arab Emirates	1	0	5	1

^a ^Case numbers reported in 2022 that are elevated vs the previous 5 years are indicated.

Probable cases (n = 111) were reported in Croatia (n = 1), Denmark (n = 4), Germany (n = 1), Italy (n = 12), Portugal (n = 3), Spain (n = 9), Sweden (n = 4), Switzerland (n = 1), the Netherlands (n = 2), UK (n = 37), Ukraine (n = 6), Colombia (n = 18), India (n = 3), Israel (n = 5), and the United Arab Emirates (n = 5) by 26 hospitals in 2022. Probable case numbers were elevated in 6 hospitals in Italy, Spain, Sweden, the UK, Ukraine, and Israel in 2022 compared with the previous years (of 20 hospitals with probable cases and data reported for previous years).

Severe cases (n = 36) were reported in Denmark (n = 2), Germany (n = 1), Italy (n = 5), Poland (n = 5), Spain (n = 5), Sweden (n = 4), the Netherlands (n = 1), UK (n = 5), Colombia (n = 4), India (n = 3), and the United Arab Emirates (n = 1) by 16 hospitals. Severe case numbers were elevated in five hospitals in Italy, Poland, Spain, Sweden, and the UK in 2022 compared with previous years (of 13 hospitals with severe cases and data reported for previous 5 years).

## Discussion

The number of paediatric cases of probable acute hepatitis of unknown aetiology seem to be elevated in 5 out of 17 surveyed European countries and 1 out of 7 surveyed non-European countries compared with previous years, with the highest case numbers reported in the UK since the beginning of this year. In the UK, Adenovirus F type 41 was detected in 18 cases with available typing data (91/126 cases (72%) tested positive for adenovirus), and has been suggested as a causative agent with or without a cofactor, i.e. increased susceptibility given lack of prior adenovirus exposure because of coronavirus disease (COVID-19) public health measures, prior or co-infection with severe acute respiratory syndrome coronavirus 2 (SARS-CoV-2) or other pathogen, or a toxin, drug, or environmental exposure [4,5]. Other suggested hypotheses include a novel pathogen, a novel variant of adenovirus, a drug, a toxin, an environmental exposure, or a novel variant of SARS-CoV-2. 

We used similar case definitions as initially used in the UK and by the World Health Organization (WHO) for comparability of results [1,6]. However, as knowledge is gained, we do suggest reassessing the case definitions as well as excluding all known causes of acute hepatitis, i.e. other well-known pathogens including EBV, HSV, intoxication, autoimmune hepatitis or metabolic syndrome, for future studies. Acute hepatitis of unknown aetiology is a rare syndrome among children as reflected by the low case numbers reported by the individual hospitals. The case numbers were too low to use statistical methods for a time trend analysis and we used an arbitrary threshold to indicate countries with an elevation in the case numbers as an alternative. The case number elevations as described in this report should therefore be interpreted as no more than a crude early warning signal. 

This study has several limitations. We could not determine the response rate since the survey link was sent to an unknown number of recipient hospitals via several clinical networks. The results of this study might have been biased towards hospitals with an unusually high number of cases. In addition, severe cases may have been counted more than once for countries with multiple reporting hospitals since we did not have unique identifiers for cases and cases may have been transferred between hospitals. Some hospitals reported difficulties in retrieving (retrospective) case data, despite the use of electronic hospital databases. In our opinion, this requires a review on how this type of data can be made available in the future in the light of epidemic and pandemic preparedness. 

## Conclusions

Our study provides a comparison of the incidence of paediatric cases of acute hepatitis of unknown origin with baseline case numbers in the previous 5 years. Further epidemiological, immunological, and clinical studies using metagenomic sequencing and other techniques, e.g. immunophenotyping, RNA expression profiling and toxicological analysis, are required to identify the aetiology, risk factors, and progression of this ongoing outbreak.
