# Author's correction for Euro Surveill. 2022;27(19)

**DOI:** 10.2807/1560-7917.ES.2022.27.20.2200370c

**Published:** 2022-05-19

**Authors:** Eurosurveillance editorial team

**Affiliations:** 1 European Centre for Disease Prevention and Control (ECDC), Stockholm, Sweden

In the article ‘Case numbers of acute hepatitis of unknown aetiology among children in 24 countries up to 18 April 2022 compared to the previous 5 years’ by Janko van Beek et al., published on 12 May 2022, the full name of ECRAID was written as The European clinical research network of infectious diseases. This was corrected to European Clinical Research Alliance on Infectious Diseases on 13 May 2022 at the request of the authors. 

